# Urinary biomarkers for hepatocellular carcinoma: current knowledge for clinicians

**DOI:** 10.1186/s12935-023-03092-5

**Published:** 2023-10-13

**Authors:** Kaige Deng, Jiali Xing, Gang Xu, Bao Jin, Xueshuai Wan, Yongchang Zheng, Shunda Du, Xinting Sang

**Affiliations:** 1 Department of Liver Surgery, Peking Union Medical College Hospital, Chinese Academy of Medical Sciences, 1 Shuaifuyuan, Dongcheng District, Beijing, 100730 China; 2 Department of Liver Surgery and Liver Transplant Center, West China Hospital of Sichuan University, Chengdu, China

**Keywords:** Hepatocellular carcinoma, Urine testing, Biomarkers, Diagnosis, Prognosis, Multi-omics

## Abstract

Hepatocellular carcinoma (HCC) is the most predominant primary liver cancer, causing many illnesses and deaths worldwide. The insidious clinical presentation, difficulty in early diagnosis, and the highly malignant nature make the prognosis of HCC extremely poor. The complex and heterogeneous pathogenesis of HCC poses significant challenges to developing therapies. Urine-based biomarkers for HCC, including diagnostic, prognostic, and monitoring markers, may be valuable supplements to current tools such as serum α-fetoprotein (AFP) and seem promising for progress in precision medicine. Herein, we reviewed the major urinary biomarkers for HCC and assessed their potential for clinical application. Molecular types, testing platforms, and methods for building multimolecule models in the included studies have shown great diversity, thus providing abundant novel tools for future clinical transformation and applications.

## Introduction

Primary liver cancer is one of the leading malignancies of the digestive system, including hepatocellular carcinoma (HCC), which accounts for the majority (75–85%) of liver cancers, followed by cholangiocarcinoma (ICC) and other rare histological types. Liver cancer is the 6th most prevalent and the 3rd most lethal cancer type. About 906,000 new cases and 830,000 new deaths were reported in 2020. The disease burden is more significant in East Asia, Southeast Asia, North Africa, and West Africa than in other regions [1]. The major risk factors for HCC include chronic hepatitis B virus (HBV) or hepatitis C virus (HCV) infection, food or water contamination of aflatoxin, and alcoholism. The main risk factors vary from region to region. In high-risk regions such as China, South Korea, and sub-Saharan Africa, chronic HBV infection, aflatoxin exposure, or both are the leading etiologies for HCC, while HCV infection may be the primary factor in other regions [1, 2]. Furthermore, the etiological spectrum of HCC is undergoing a shift, i.e., a decline in the prevalence of hepatitis and the increase in HCC burden caused by overweight, diabetes, nonalcoholic fatty liver disease (NAFLD), and nonalcoholic steatohepatitis (NASH) [3]. Therefore, traditionally high-risk countries such as China, which have gained huge benefits from preventing and controlling HBV, face novel challenges [4].

HCC is a highly aggressive malignancy with insidious and non-specific clinical manifestations. Therefore, cases are mostly at advanced stages when diagnosed, leading to a limited prognosis. In China, the 5-year age-standardized net survival rate of liver cancer patients from 2010 to 2014 was only 14.1% [5]. Currently, surgical intervention is the primary modality of treatment for HCC patients to acquire long-term survival. However, survival benefits significantly rely on clinical staging. According to a study based on 10,996 Chinese patients with HCC treated with surgery between 2009 and 2019, the 5-year survival rate of patients with advanced tumors is only 23.8%, which is < 1/3rd of those with early tumors [6]. Thus, early detection of HCC can preserve the liver function reserve and provide several therapeutic options [6, 7]. Although the diagnostic tools have improved, the un-specific and diverse biological behavior hinders the early detection of HCC [8].

Tumor markers from plasma or serum have been widely explored and used, but HCC lacks reliable biomarkers. For example, the sensitivity and specificity of the most commonly used tumor marker, serum AFP, is insufficient. The sensitivity of AFP ranges from 39 to 65%, while the specificity ranges from 79 to 94%, depending on different cutoff values [9]; the sensitivity for early HCC is only 32–49% [10]. AFP also shows an elevation in benign lesions such as hepatitis and cirrhosis [11]. Various guidelines no longer recommend AFP alone as a diagnostic test; rather, the combination of screening or diagnostic imaging studies, such as ultrasound, computed tomography (CT), and magnetic resonance imaging (MRI), are required [12, 13]. As a result, the current diagnostic algorithms for HCC are constrained by inadequate equipment and professional staff; this situation is pronounced in developing regions with a heavy burden of HCC. Therefore, there is an urgent need for simple and easy testing methods as well as accurate and reliable biomarkers to reducing the mortality of HCC.

Urine testing is a noninvasive method widely studied as an indicator of the state of health, and the specimens can be collected, transported, and stored easily [14]. As an ultrafiltrate of blood, urine accumulates abnormal waste products from circulation to maintain homeostasis, including markers of early oncogenesis, which might be more abundant and detectable than markers from blood [15, 16]. In addition, since urine does not maintain a homeostatic environment like blood, the urine samples are resistant to environmental changes and less likely to be disturbed or contaminated during the examination procedures [15]. The total abundance of proteins, nucleic acids, and other molecules is lower in urine than in blood, further facilitating the accurate identification of the biomarkers due to the lower signal-to-noise ratio. In recent years, there has been an increasing trend for studies that identify HCC biomarkers from the urine; some of these markers have shown promising value in the diagnosis, treatment, monitoring, and prognosis of HCC [8, 16–21]. In addition, multi-omics approaches that allow high-throughput comprehensive profiling of urine samples are also gaining popularity [14]. The clinical transformation of these findings has great significance in improving the management of HCC and the prognosis of patients.

This review aimed to summarize the progress in urine-based biomarkers for HCC to improve the clinicians’ understanding of cutting-edge discoveries and develop novel biomarkers to improve HCC management.

## Testing of urine samples

**Fig. 1:**
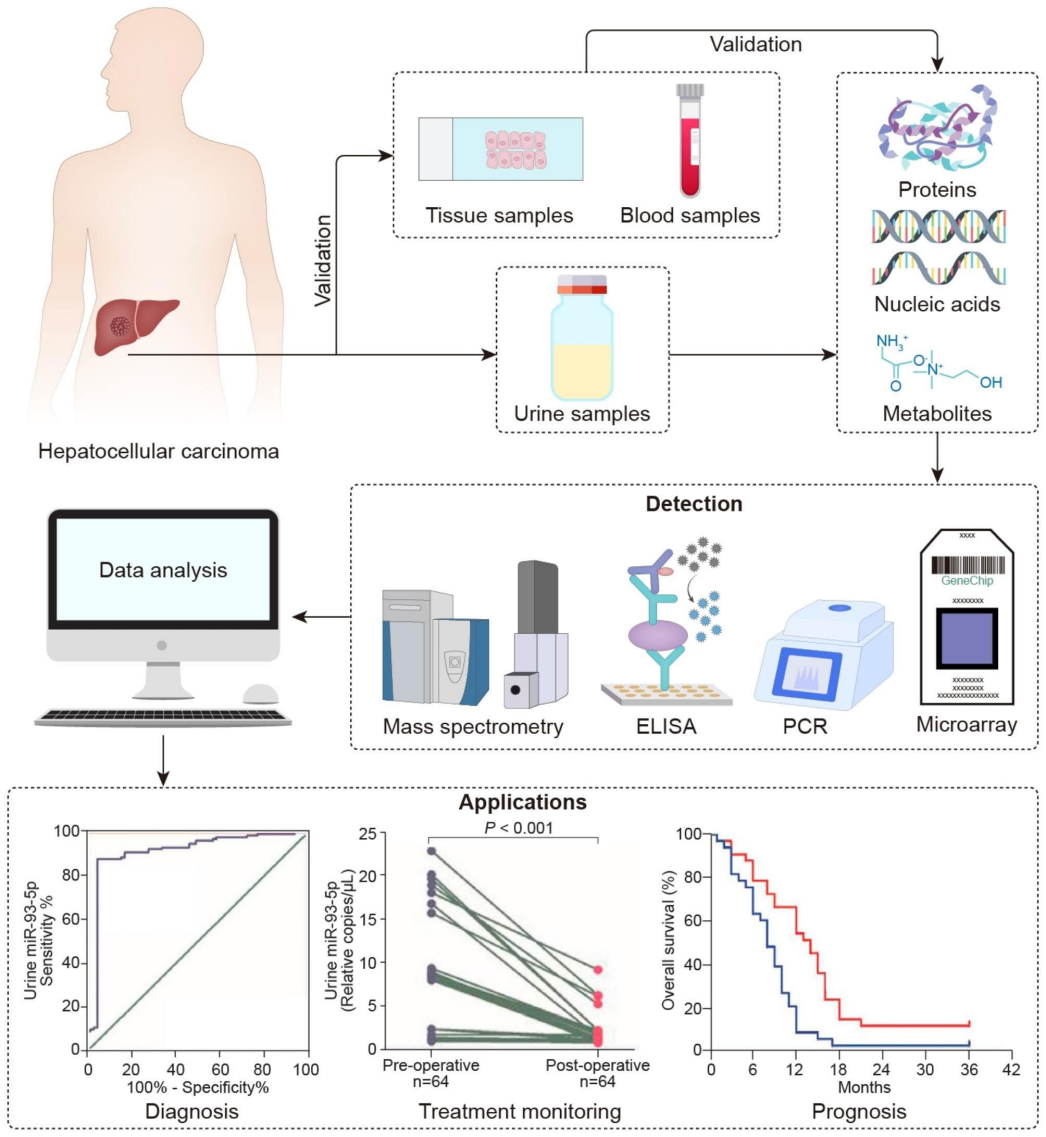
Urinary biomarkers for HCC: the origins, testing platforms, and applications. The components of the urine samples, including proteins, nucleic acids, and metabolites, are tested and screened for biomarkers of HCC with indicative value in the diagnosis, prognosis, and treatment monitoring of HCC

The origin, detection, and application of urinary biomarkers for HCC are illustrated in Fig. 1. In terms of composition, urinary biomarkers for HCC include products derived from each level of the Central Dogma and the downstream physiological and pathological processes involving DNA, RNA, proteins, and metabolites [14]. These molecules in urine require several common characteristics. First, a small molecular weight (≤ 20 kDa) and appropriate electric charge are essential since most of these markers are produced pre-renally and filtered into the urine via the kidney. Second, the markers should be cancer-specific rather than dependent on the changes in homeostasis. Finally, a sufficient concentration of the markers in the urine is required for reliable detection [22].

**Fig. 2:**
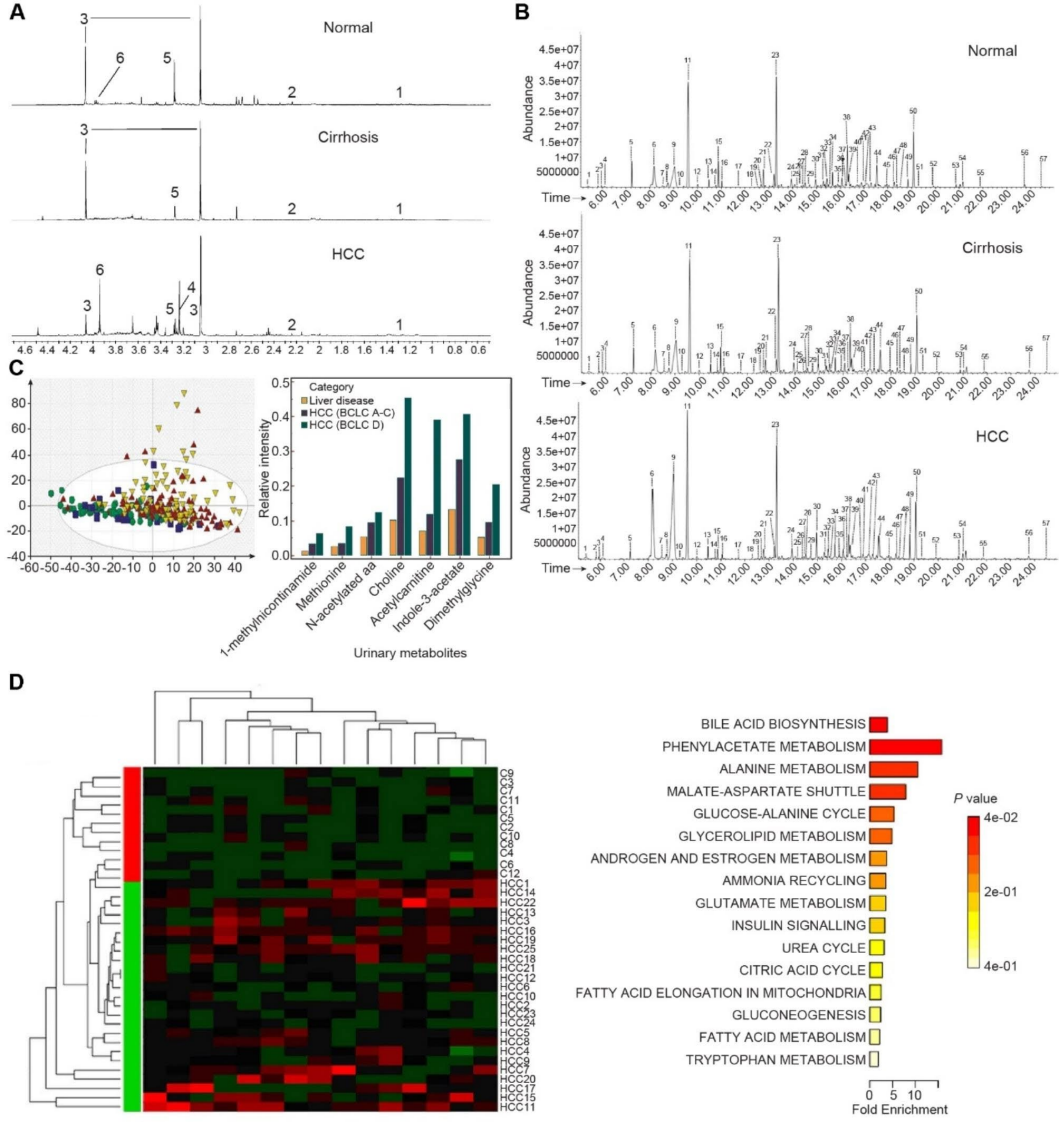
Representative multi-metabolite models in HCC. (**A**) Typical original results from testing platforms [1] H-NMR analysis of urine samples, Reprinted from *Shariff et al., 2010*. (**B**) Typical original results from testing platforms GC-MS/MS analysis of urine samples. Reprinted from *Osman et al., 2017*. (**C**) Alterations in urinary metabolic profiles from non-cirrhosis liver disease to liver cirrhosis and HCC (left) Distinct metabolomic profiles of HCC, cirrhosis, liver disease, and normal control illustrated by the PCA score plot. (right) Correlation between levels of urinary metabolites and disease categories and clinical stages of HCC. Reprinted from *Ladep et al., 2014*. (**D**) Differential metabolites and altered metabolic pathways between HCC and normal control. (left) Metabolomic alterations in HCC compared to normal controls illustrated by heatmap. (right) Major dysregulated pathways in HCC are illustrated by pathway-associated metabolite set enrichment analysis. Reprinted from *Liang et al., 2016*

Various types of testing platforms are used for different markers. Radioimmunoassay (RIA) and enzyme-linked immunosorbent assay (ELISA) are commonly used for the quantitative determination of proteins and metabolites, while DNA and RNA markers are quantified by polymerase chain reaction (PCR). With recent advances in detection tools, the throughput, sensitivity, and accuracy of urinary molecular tests have been improved markedly, facilitating a comprehensive screening of tumor markers in urine. For example, proton nuclear magnetic resonance ( [1] H-NMR) and gas or liquid chromatography-mass spectrometry (GC-MS/MS or LC-MS/MS) facilitate high-throughput quantification of urinary metabolites or proteins (Fig. 2A, B), whereas microarray and next-generation sequencing (NGS) supports extensive screening of urinary nucleic acids. Additionally, a variety of machine learning algorithms, including logistic regression (LR), principal component analysis (PCA), partial least squares discriminant analysis (PLS-DA), and random forest (RF), are utilized to build multi-molecule models [23].

**Fig. 3:**
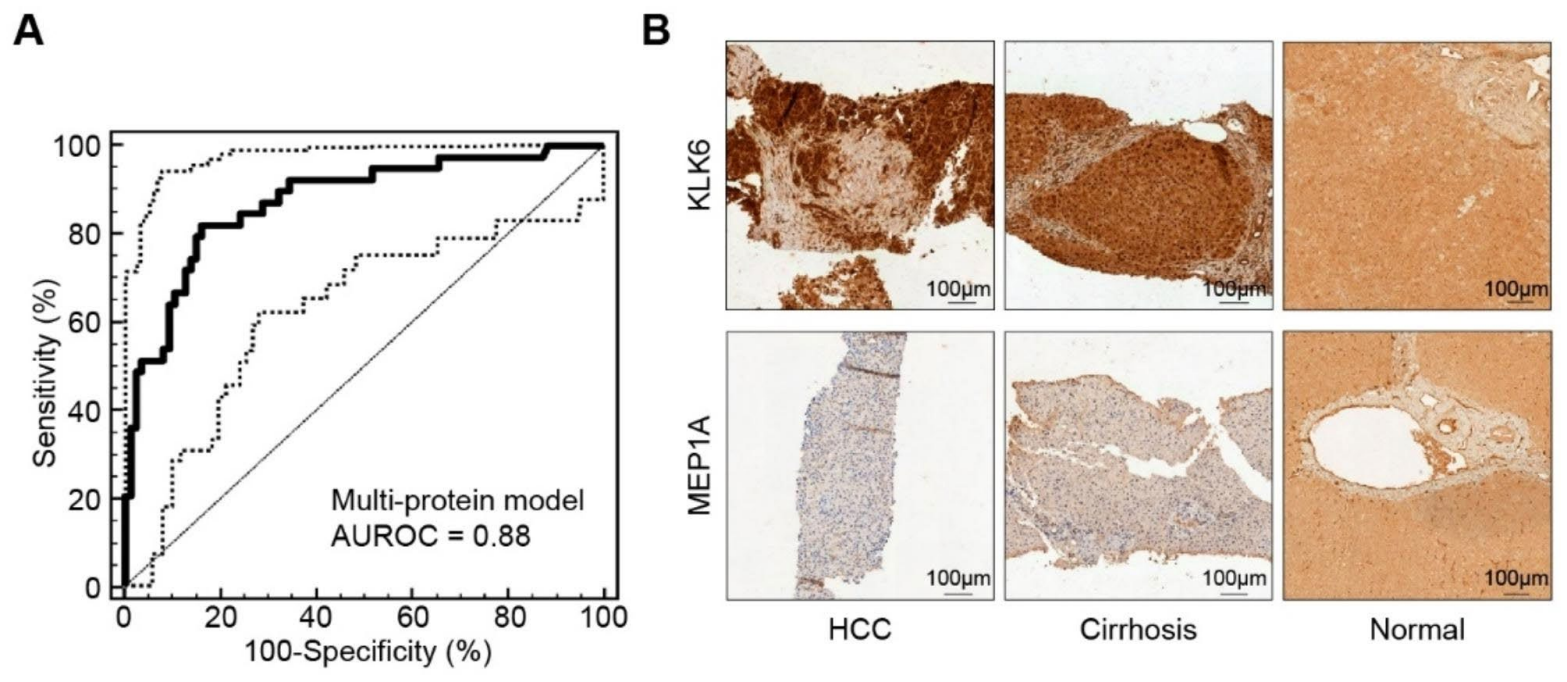
A combined analysis of urinary proteomics and tissue IHC. (**A**) Diagnostic power of a urinary proteomic model including 31 peptide markers for HCC, illustrated by ROC. (**B**) Tissue IHC confirmed the dysregulation of KLK6 and MEP1A, two proteases potentially involved in HCC progression, deduced by the N- and C-terminals of 31 differential peptides. (A–B) Reprinted from *Bannaga et al., 2017*

**Fig. 4:**
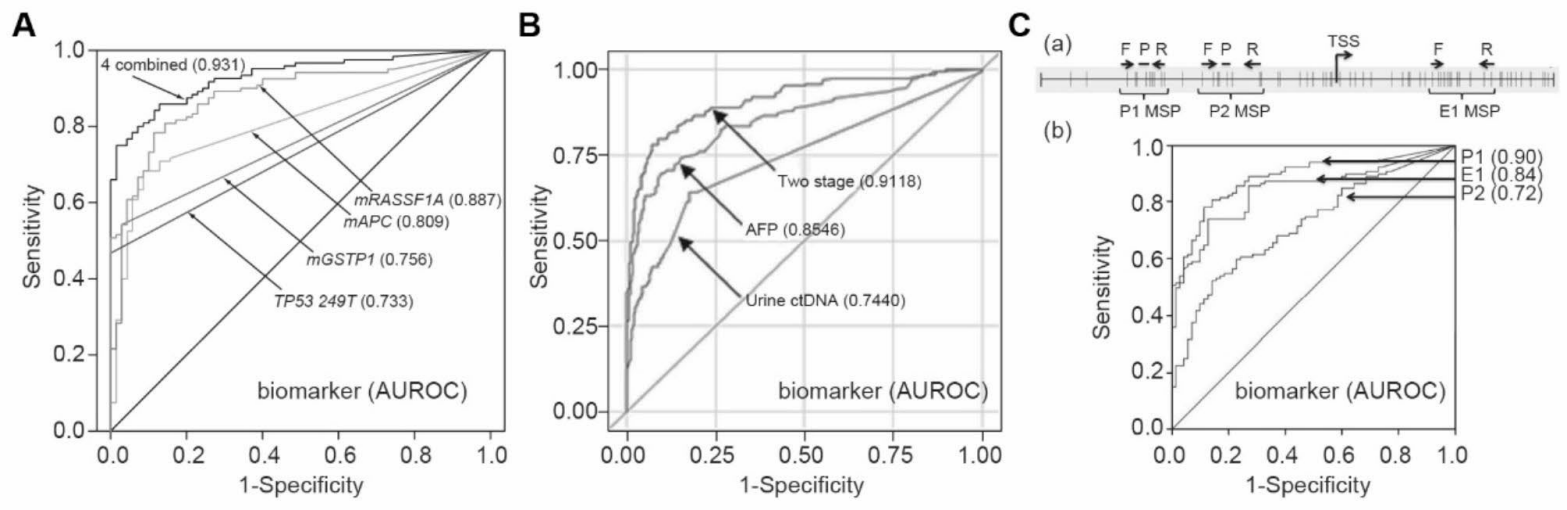
Representative urinary ctDNA biomarkers for HCC. (**A**) Diagnostic performance of multi-ctDNA marker panel for HCC. Reprinted from *Su et al., 2014*. (**B**) A two-stage model combining ctDNAs and serum AFP in the diagnosis of HCC. Reprinted from *Kim et al., 2022*. (**C**) Improving the specificity of urinary ctDNA marker *mRASSF1A* by detecting the methylation at different sites. (a) Different methylation sites in the promoter and first exon of *RASSF1A* gene. (b) Methylation of P1 is the most specific HCC marker among the three types of *mRASSF1A*, with the highest AUROC. Reprinted from *Jain et al., 2015*

**Fig. 5:**
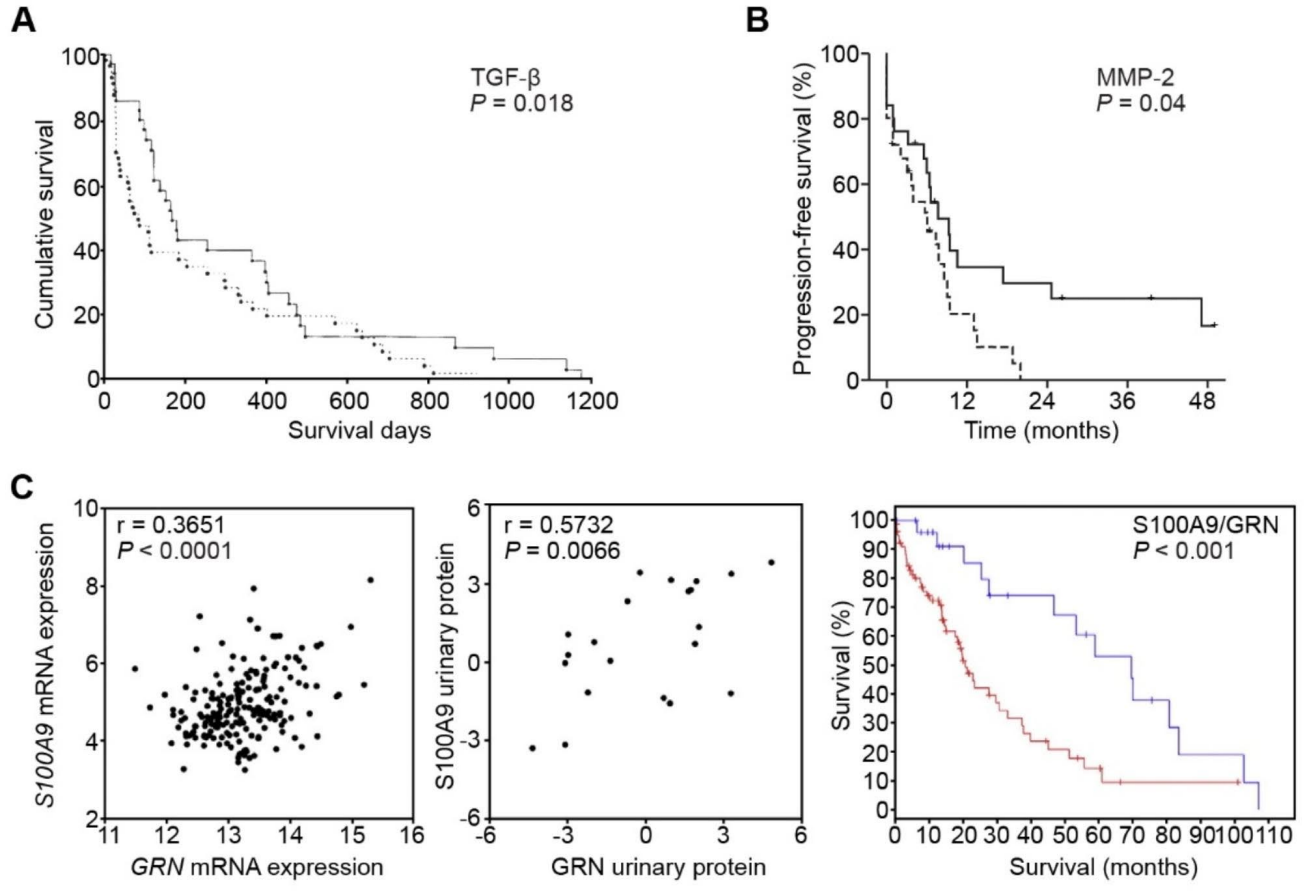
Representative urinary protein biomarkers for HCC. (**A**) Prognostic value of urinary protein TGF-β1 in HCC patients illustrated by Kaplan–Meier plots. Reprinted from *Tsai et al., 1997*. (**B**) Prognostic value of urinary protein MMP-2 in HCC patients as illustrated by Kaplan-Meier plots. Reprinted from *Suh et al., 2014*. (**C**). Prognostic value of urinary multiprotein models in HCC patients. (left) Co-expression of *S100A9* and *GRN* mRNA in tumor tissues. (middle) Associated elevations in urinary S100A9 and GRN proteins. (right) Prognostic value of both S100A9 and GRN amplification/gain in HCC patients. Reprinted from *Huang et al., 2015*

**Fig. 6:**
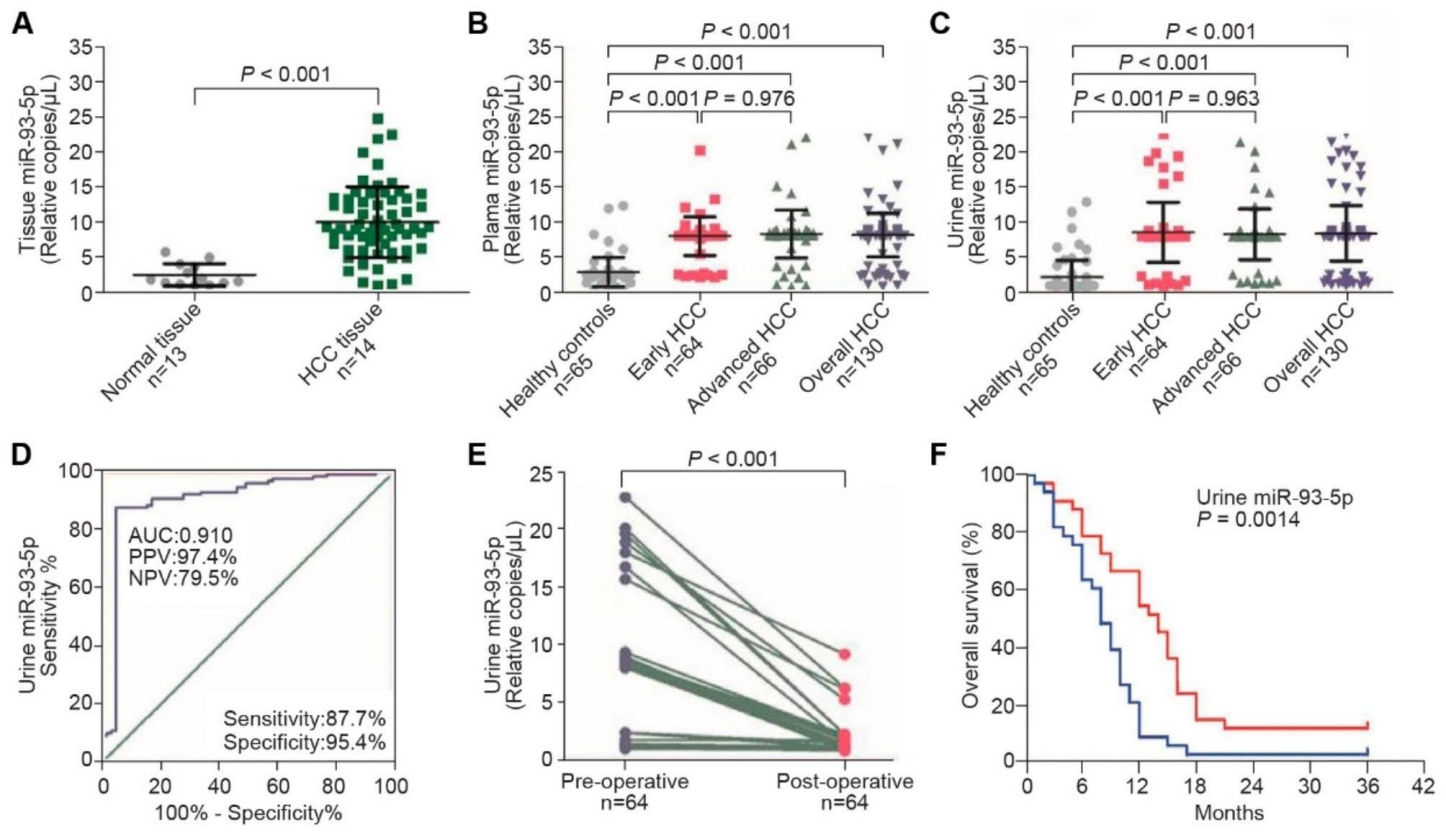
Representative urinary microRNA marker for HCC. (**A**) The consistent upregulation of miR-93-5p in tissue with HBV-related HCC. (**B**) The consistent upregulation of miR-93-5p in plasma in HBV-related HCC. (**C**) The consistent upregulation of miR-93-5p in urine in HBV-related HCC. (**D**) Application of urinary miR-93-5p in the detection of HBV-related HCC. (**E**) Application of urinary miR-93-5p in the treatment monitoring of HBV-related HCC. (**F**) Application of urinary miR-93-5p in the prognosis of HBV-related HCC. (A–F) Reprinted from *Zhou et al., 2022*

The clinical applications of the reviewed biomarkers are primarily to aid in the diagnosis, prognostic assessment, or monitoring of treatment response of HCC. Sensitivity, specificity, and area under the receiver operating characteristic curve (AUROC) are the main parameters for evaluating diagnostic efficacies (Figs. 3A and 4A–C). Kaplan-Meier survival analysis is commonly used to judge the stratification power for survival outcomes (Figs. 5A-C and 6F). The correlations with well-established prognostic indicators, such as pathological tumor features and clinical stages, also reflect the prognostic efficacy of urinary markers. Also, some markers have shown potential in predicting HCC risk in community populations, evaluating the treatment response, and predicting recurrence.

The information on the included studies is described in Tables 1 and 2. Notably, there is an increasing trend of multi-molecule model studies that might overcome the intra- and inter-tumor heterogeneity of HCC compared to single-molecule biomarkers, especially when the model components belong to distinct cancer signaling pathways [23, 24].

**Table 1:** Brief information about non-metabolomics studies on urinary biomarkers for hepatocellular carcinoma

Author/Year	Nations*	Samples	Platforms	Modeling	Validation	Categories	Biomarkers	Applications ^a^	Results	Evidence from Serum/tissue	Serum AFP
**Urinary proteins**											
Yeh/ 1987	Taiwan, China	HCC, 31 Probably HCC, 15 Healthy, 21	RIA			TGF	TGF-α↑	Diagnosis	(Probably) HCC vs. Healthy Cutoff at 10.5 μg/g creatinine Sensitivity 71.7%		Cut-off at 400ng/ml Sensitivity 58.7% [TGF-α + AFP] Sensitivity 93.5%
Tsai/ 1997	Taiwan, China	HCC, 94 LC, 94 Healthy, 50	RIA			TGF	TGF-β1↑	Diagnosis	HCC vs. LC AUROC 0.730 Cutoff at 50 μg/g creatinine Sensitivity 53.1% Specificity 98.9%		AUROC 0.730 Cut-off at 100ng/ml Sensitivity 55.3% Specificity 98.9% OR 1.06 (1.02–1.10) [TGF-β1 + AFP] Sensitivity 84.0% Specificity 97.8%
								Independent risk factor	OR 1.08 (1.04–1.12)^b^		
								Prognosis (Pathological features)	Correlation with diffuse HCC (P = 0.001), larger tumors (≥ 3 cm, P = 0.018), more Child-Pugh C (P = 0.026).		
Tsai/ 1997	Taiwan, China	HCC, 94 LC, 94	RIA			TGF	TGF-β1↑	Prognosis (Monitor)	After TACE TGF-β1↓ (P = 0.0001)		
								(OS)	TGF-β1↑ vs. TGF-β1 normal OS↓ (P = 0.018)		
Noie/ 2001	Japan	Partial hepatectomy, 61 HCC, 40	RIA			UTI	UTI ^c^	Other	Postoperative ΔuUTImax correlated with: ICG clearance rate (P = 0.002) Operation duration (P = 0.034) Resection rate (P = 0.004)		
Lin/ 2004	Japan	HCC, 39 LC, 19 CH, 16	ELISA			UTI	UTI*	Other	HCC vs. CH, no significant difference Child-Pugh C vs. Child-Pugh A/B ↓ (P < 0.05/0.01)	HCC vs. LC (P < 0.05) LC vs. CH vs. NC↓ (P < 0.05) Child-Pugh C vs. Child-Pugh A/B ↓ (P < 0.05/0.01)	
Abdelsameea/ 2020	Egypt	HCC, 40 LC, 40 CH, 40 Healthy, 40	ELISA			NGAL	NGAL↑	Diagnosis	HCC vs. LC AUROC 0.95 Cutoff at 1255 ng/mL Sensitivity 90% Specificity 87.5%	Tissue: (Zhang/2012) Expression of NGAL/NGALR↑ (P < 0.05) correlates with: Vascular invasion (P = 0.03) TNM stage (P = 0.004) Recurrence (P < 0.001) Shorter OS (P < 0.001)	AUROC 0.92 Cut-off at 39.6ng/ml Sensitivity 85% Specificity 100% [NGAL + AFP] AUROC 0.997 Sensitivity 95% Specificity 100%
Suh/ 2014	South Korea	HCC, 50 (Radiotherapy)	ELISA			MMP	MMP-2↑	Prognosis (PFS)	MMP-2 ≥ median → worse PFS (P = 0.04) MMP-2 or serum VEGF/plt ≥ median → worse PFS OR 2.12 (1.01–4.55)		
Abdalla/ 2012	Egypt	HCC, 32 HCV, 74 Normal, 12	LC-MS/MS, RT-qPCR			Proteomics RNA	DJ-1↑, CAF-1↑, HSP60↑	Diagnosis	Urinary RNA over-expression of *CAF-1* Sensitivity 66% Specificity 90% Accuracy 78% *HSP60* Sensitivity 83% Specificity 42% Accuracy 62% [*CAF-1* + *HSP60*] Sensitivity 61% Specificity 92% Accuracy 77%		
Huang/ 2015	Taiwan, China	HCC, 44 Healthy, 44	NanoLC-MS/MS			Proteomics	S100A9↑, GRN↑	Diagnosis	S100A9, GRN peptide quantification ratio (D/H) > 1.5 Co-up-regulation (r = 0.5732, P = 0.0066)	Tissue: Gene: *S100A9* amplified in 70% HCC, *GRN* amplified in 27% HCC, *S100A9*/*GRN* co-amplification (p = 0.001) mRNA: *S100A9/GRN* co-expression (r = 0.3651, p < 0.0001) Co-amplification → worse OS (p < 0.001)	
Zhao/ 2020	China	Training set HCC, 36 LC, 29 CH, 14 Test set HCC, 18 LC, 10 CH, 9 MRM set HCC, 20 LC, 15 CH, 5	LC-MS/MS	RF	Cross-validation External validation MRM	Proteomics	HPX↑, APOH↑, APCS↑, PLG↑, GOT1↓, GLR↓, NCR3LG1↓	Diagnosis	MRM validation: HCC vs. LC/CH AUROC 0.95 Sensitivity 90.0% Specificity 85.0%		HCC vs. LC/CH AUROC 0.56
Zhan/ 2020	China	Training set HCC, 10 LC, 10 CH, 10 Healthy, 10 Validation set HCC, 75 LC, 51 CH, 14	LC-ESI-MS/MS, WB, ELISA		External validation	Proteomics	AFP↑, ORM1↑	Diagnosis	HCC vs. non-HCC AFP AUROC 0.795 (0.704–0.886) Sensitivity 63.5% Specificity 95.4% ORM1 AUROC 0.705 (0.604–0.807) [AFP + ORM1] AUROC 0.864 (0.791–0.937) Sensitivity 80.9% Specificity 85.5%		HCC vs. non-HCC AUROC 0.818 (0.734–0.903) Cutoff at 334.3 ng/ml Sensitivity 62.5% Specificity 93.8%
Bannaga/2021	UK	Training set HCC, 18 Non-HCC, 51 Validation set HCC, 39 Non-HCC, 87	CE-MS/MS, IHC	SVM	External validation	Proteomics	KLK6↑, MMP-3↑, MMP-13↑, CTSD↑, CTSE↑, MEP1A↓, CTSB↓	Diagnosis Prognosis (OS)	HCC-31 model (31 components) AUROC 0.88 (0.81–0.93) Sensitivity 79.5% Specificity 85.1% OS: OR 4.1 (1.7–9.8 P = 0.0005)	Tissue IHC: KLK6↑ MEP1A↓	
**Urinary nucleic acids**											
Lin/ 2011	Taiwan, China	HCC, 17 Healthy, 15	LNA clamp-mediated PCR			DNA	*TP53 249T*↑	Diagnosis	Sensitivity 52.9% (9/17) Specificity 100% (15/15)		
Jain/ 2015	USA Taiwan, China	HCC, 78 LC, 50 CH, 45	BS-qPCR, qMSP			DNA	*mRASSF1A*↑	Diagnosis	*mRASSF1A* at P1 HCC vs. LC/CH AUROC 0.705 HCC vs. CH AUROC 0.831 HCC vs. LC AUROC 0.595	Tissue: *mRASSF1A* at P1/E2/P2 HCC vs. LC/CH P1 AUROC 0.90 At 90%sensitivity Specificity P1 72.9% E1 38.6% P2 27.1%	In AFP (-) HCC patients 81.8% (36/44) *mRASSF1A* at P1 (+)
Hann/ 2017	USA	HCC, 10 Recurrent, 5	BS-qPCR			DNA	*mRASSF1A*↑, *mGSTP1*↑, *TP53 249T*↑	Prognosis (Recurrence)	MRI-confirmed recurrent cases: *TP53 249T* / *mRASSF1A* / *mGSTP1* (+) (up to 9 months before MRI confirmation)		MRI-confirmed recurrent cases: 3/5 AFP (-)
Zhang/ 2018	USA Taiwan, China	HCC, 97 Non-HCC (CH, LC), 112	qPCR			DNA	*TP53 249T*↑, *CTNNB1 32–37*↑, *hTERT 124*↑, *mRASSF1A*↑	Diagnosis	[*TP53 249T + hTERT 124 + mRASSF1A*] HCC vs. non-HCC AUROC 0.607 Sensitivity 26.2% Specificity 85.7%	Serum [*TP53 249T + hTERT 124 + mRASSF1A*] HCC vs. non-HCC AUROC 0.846 Sensitivity 76.2% Specificity 85.7%	HCC vs. non-HCC AUROC 0.799 Sensitivity 71.4% Specificity 81.0% [AFP + urine + serum] AUROC 0.904 Sensitivity 90.5% Specificity 81.0%
Wang/ 2018	Taiwan, China	HCC, 137 CH, 224 LC, 207	Not specified	LR, CART, FS (serum AFP→LR), RF, TS (LR→FS)	Cross-validation	DNA	*mRASSF1A*↑, *mGSTP1*↑, *TP53 249T*↑	Diagnosis	[serum AFP + ctDNA] (TS) AUC 0.935 (0.930–0.940) Sensitivity 87.9% Specificity 90%		AUROC 0.88 Sensitivity 48.2% Specificity 90%
Kim/ 2022	USA Taiwan, China	HCC, 186 LC, 144 CH, 279	qPCR	TS (AFP→LR)	Cross-validation	DNA	*TP53 249*↑, *mRASSF1A*↑, *mGSTP1*↑	Diagnosis	[ctDNA panel] AUROC 0.715 (0.668–0.762) [serum AFP + ctDNA] (TS) AUROC 0.902 (0.871–0.933) Sensitivity 90% Specificity 79.6% BCLC-A HCC Sensitivity 90% Specificity 77%		AUROC 0.8546 (0.8184–0.8908) BCLC-A HCC Sensitivity 90% Specificity 40%
Abdalla/ 2012	Egypt	HCC, 32 HCV-positive, 74 Normal, 12	MicroRNA array RT-qPCR			RNA	miR-625↑, miR-532↑, miR-618↑, miR-516-5p↓, miR-650↓	Diagnosis	HCC vs. HCV-positive [miR-618 + miR-650] Sensitivity 58% Specificity 75% Accuracy: 69%		
Świtlik/ 2019	Poland	HCC, 65 Healthy, 29	MicroRNA array RT-qPCR	Exploratory factor analysis		RNA	miR-618↑, miR-532-3p↑, miR-625↑, miR-640↑, miR-765↑	Diagnosis	[miR-532-3p + miR-765] HCC vs. Healthy: Wilks χ [2] = 0, P < 0.0001	Different miRNA profiles from tissue/serum/facet	
								Prognosis (Pathological features)	[miR-532-3p + miR-765] clustering Correlate with histological grade, clinical stage, classification for primary tumor, lymph node, and distant metastasis P < 0.005		
Zhou/ 2022	China	Early-HCC, 64 Advanced-HCC, 66 Healthy, 65	RT-qPCR	GEO2R http://www.ncbi.nlm.nih.gov/geo/geo2r/)		RNA	miR-93-5p↑	Diagnosis	Advanced HCC vs. early HCC vs. Healthy 3.6-fold↑, 3.7-fold↑ Early HCC vs. Healthy Sensitivity 87.5% Specificity 97.4%	Tissue miR-93-5p HCC vs. non-HCC 4.0-fold↑ Serum miR-93-5p HCC vs. non-HCC Advanced HCC vs. early HCC vs. Healthy 2.9-fold↑, 2.8-fold↑ Early HCC vs. Healthy Sensitivity 85.9% Specificity 95.4%	
								Prognosis (Monitor)	1 month after hepatectomy HCC vs. non-HCC Not significant		
								(OS)	miR-93-5p↑ vs. miR-93-5p normal OS↓ (Early HCC, P = 0.0031) miR-93-5p↑ vs. miR-93-5p normal OS↓ (Advanced HCC, P = 0.0014)		
**Urinary metabolites**											
Antoniello/ 1998	Italy	HCC, 16 LC, 32 Healthy, 28	HPLC			Polyamines	PUT↑, SPM↑, SPD↑	Diagnosis	PUT (total, free, monoacetylated) ↑ (P < 0.001) SPM (total, free) ↑ (P < 0.001) SPD (total, free, monoacetylated, N^1^/N^8^ ratio) ↑ (P < 0.001)		
Enjoji/ 2004	Japan	HCC, 53 LC, 50 CH, 89 FL, 22	ELISA			Polyamines	DiAcSPM↑	Diagnosis	Cutoff at 325 nM/g creatinine Sensitivity 65.5% Specificity 76.0%		Cutoff at 20 ng/ml Sensitivity 63.8%
								Prognosis (Monitor)	Treated HCC, DiAcSPM↓ (P = 0.0431) Untreated HCC, DiAcSPM↑		
Liu/ 2013	China	Hepatic cancer, 20 Healthy, 20	UHPLC-MS/MS			Polyamines	NSPD↑, SPM↑, SPD↑	Diagnosis	Cancer vs. Normal P < 0.05↑	Serum PUT, SPD↑; L-ornithine, γ-aminobutyric acid↓	
Yu/ 2015	Animal (Rats)	HCC model, 40 Treated, 20 Normal, 20	UHPLC-MS/MS			Polyamines	NSPD↑, NSPM↑, DiAcSPD↑, DiAcSPM↑	Diagnosis	HCC vs. Normal P < 0.05↑	Tissue PUT↑ Serum NSPD↑ Serum/Tissue/Urine NSPD↑	
								Prognosis (Monitor)	Treated vs. HCC P < 0.05↓		
Dusheiko/ 1982	South Africa	HCC, 31 Other malignancies, 16 Liver dysfunction, 16 Healthy, 25	RIA			Nucleotides	cGMP↑	Diagnosis	HCC, liver dysfunction, other malignancies vs. Healthy ↑ (P < 0.0005) Cutoff at 0.95 nmol/100 mL GF Sensitivity 80% in HCC, 68% in other malignancies, 75% in liver dysfunction	cGMP↑ in HCC and liver dysfunction	
Sakai/ 1990	Japan	Mixed, including Hepatic cancer, 41 LC, 21	Biochemistry			L-Fucose	L-Fucose↑	Diagnosis	Cutoff at 215 μmol/g creatinine Hepatic cancer: Sensitivity 90.5% (19/21) LC: Sensitivity 85.4% (35/41) Gastric cancer, lung cancer, and gallbladder cancer ↑		
Bannaga/ 2021	UK	HCC, 31 Prostate cancer, 62 Bladder cancer, 29 Non-cancer, 18	SPME	PCA RBFN	50% validation set	VOCs	Not specified	Diagnosis	In AFP ≥ 10 kU/L Sensitivity 83% AUC 0.83 (0.73–0.93) In AFP < 10 kU/L Sensitivity 68% AUC 0.68 (0.54–0.81)		Serum AFP alone (cutoff 10 ku/L) Sensitivity 54.8%
Bannaga/ 2021	UK	HCC, 20 Healthy/NAFLD, 38	GC-IMS GC-TOF-MS/MS	RF LR		VOCs	2-Butanone↑, 6 other VOCs↓	Diagnosis	Significantly different in HCC vs. Non-HCC GC-IMS model (factors not specified): HCC vs. LC AUROC 0.97 (0.91–1.00) Sensitivity 0.43 (0.13–0.75) Specificity 0.95 (0.86–1.00)		
**Etiology/carcinogenesis-related biomarkers**											
Ross/ 1992	China	HCC, 22 Healthy, 140	HPLC			Aflatoxin-related	AFP_1_↑, AFB_1_-N [7]-Gua↑, AFM_1_↑, AFB_1_↑	Independent risk factor	Any of the compounds RR 3.8 (1.2–12.2)		
Wang/ 1996	Taiwan, China	HCC, 56 Healthy, 220	ELISA			Aflatoxin-related	Aflatoxin metabolites (mainly AFB_1,_ cross reactivity with AFB_2_, AFM_1_, AFG_1_, AFP_1_ etc.)↑	Independent risk factor	1st half OR 3.8 (1.1–12.8) 1st tertile OR 7.2 (1.5–34.3)	Serum aflatoxin-albumin adducts↑	
Hatch/ 1993	Taiwan, China	Residents, 250	ELISA			Aflatoxin-related	Aflatoxin metabolites (mainly AFB_1,_ cross reactivity with AFB_2_, AFM_1_, AFG_1_, AFP_1_ etc.)↑	Independent risk factor	Individual biomarker level & Area HCC mortality: positive correlation P < 0.05		
Nair/ 2004	Thailand	AC, unknown CH, unknown LC, unknown HCC, unknown	Immuno-enriched HPLC- fluorescence			Oxidative stress-related	ε-dA↑	Diagnosis	[CH, LC, HCC] vs. [AC] 20-90-fold↑		
Wu/ 2008	Taiwan, China	HCC, 74 Healthy, 290	ELISA			Oxidative stress-related & Aflatoxin-related	15-F_2t_-IsoP↑, 8-oxodG↑, AFB_1_↑	Independent risk factor	AFB_1_ correlates with 8-oxodG and 15-F_2t_-IsoP (P < 0.0001) 15-F_2t_-IsoP: 1st half OR 2.53 (1.30–4.93) 1st tertile OR 6.27 (2.17–18.13) 2nd tertile OR 3.87 (1.32–11.38)		
Ma/ 2018	China	HCC, 363 Healthy, 725	ELISA			Oxidative stress-related	15-F_2t_-IsoP↑	Independent risk factor	4th quartile vs. 1st quartile Male OR 8.84 (2.74–28.60) Female OR 1.75 (0.70–4.42)		
Yuan/ 2019	China	HCC, 347 Healthy, 691	LC-ESI-MS/MS			Oxidative stress-related	8-epi-PGF2α↑	Independent risk factor	4th quartile vs. 1st quartile OR 5.29 (1.92–14.54)		

^a^ Only include the application of urinary biomarkers. ^b^ Ranges in parentheses represent the 95% confidence interval (95% CI). ^c^ Parameters that cannot be classified as up- or downregulated in hepatocellular carcinoma/liver cancer. * Nations refer to the countries/regions of the tested patients, studies in animals are shown as the species of aexperimented animals

**Samples**: HCC, hepatocellular carcinoma. LC, liver cirrhosis. CH, chronic hepatitis. MRM, multiple reaction monitoring. HCV, hepatitis C virus. FL, fatty liver. **Platforms**: RIA, radioimmunoassay. ELISA, enzyme-linked immunosorbent assay. LC-MS/MS, liquid chromatography-tandem mass spectrometry. LC-ESI-MS/MS, liquid chromatography-electrospray ionization-tandem mass spectrometry. WB, western blot. RT-qPCR, quantitative reverse transcription PCR. CE-MS/MS, capillary electrophoresis mass spectrometry. IHC, immunohistochemistry. LNA, locked nucleic acid. BS-qPCR, bisulfite quantitative PCR. qMSP, quantitative methylation-specific PCR. UHPLC-MS, ultra-high-performance liquid chromatography-tandem mass spectrometry. HPLC, high-performance liquid chromatography. GC-TOF-MS/MS, gas chromatography–time-of-flight mass spectrometry. GC-IMS, gas chromatography–ion mobility spectrometry. SPME, solid-phase microextraction. **Modeling and validation methods**: RF, random forest. SVM, support vector machine. LR, logistic regression. CART, classification and regression trees. FS, fixed sequential. TS, two-step. **Categories and biomarkers**: TGF, transforming growth factor. UTI, urinary trypsin inhibitor. NGAL(R), neutrophil gelatinase-associated lipocalin (receptor). MMP, matrix metalloproteinase. HPX, hemopexin. APOH, apolipoprotein H. APCS, amyloid P component, serum. PLG, plasminogen. GOT1, glutamic oxaloacetic transaminase 1. GLRX, glutaredoxin. NCR3LG1, natural killer cell cytotoxicity receptor 3 ligand 1. CAF-1, chromatin assembly factor-1. HSP60, heat shock protein 60. KLK6, kallikrein-6. CTS, cathepsins. MEP1A, meprin A subunit α. PUT, putrescine. SPM, spermine. SPD, spermidine. NSPM, N-acetylspermine. NSPD, N-acetylspermidine. DiAcSPM, N [1], N [12]-diacetylspermine. DiAcSPD, N [1], N [8]-diacetylspermidine. AF, aflatoxin. Gua, guanine. ε-dA, etheno-deoxyadenosine. 8-epi-PGF2α, 8-epi-Prostaglandin F2α. 15-F_2t_-IsoP, 15-F2t-isoprostane. 8-oxodG, 8-oxo-7,8-dihydro-2’-deoxyguanosine. **Results**: AFP, alpha-fetoprotein. AUROC, area under the receiver operating characteristic. TACE, transarterial chemoembolization. ICG, indocyanine green. CRP, C reactive protein. OS, overall survival. PFS, progression-free survival. OR, odds ratio. BCLC, Barcelona Clinic Liver Cancer. GF, glomerular filtration

**Table 2:** Brief information about metabolomics studies on urinary biomarkers for hepatocellular carcinoma

Author/Year	Nations*	Samples	Platform	Modeling	Validation	Biomarkers	Applications ^a^	Results	Evidence from Serum/tissue	AFP Comparison
Shariff/ 2010	Nigeria	HCC, 18 LC, 10 Healthy, 15	[1] H-NMR	PCA PLS-DA	Cross-validation External validation (30% samples)	carnitine↑, creatine↑, creatinine↓, acetone↓	Diagnosis	HCC vs. Healthy Sensitivity 100% Specificity 93.3% HCC vs. LC Sensitivity 89.5% Specificity 88.9%		Cutoff at 20 IU/mL Sensitivity 88.9% Specificity 77.8%
Shariff/ 2011	Egypt	HCC, 18 LC, 20 Healthy, 20	[1] H-NMR	PCA PLS-DA	Cross-validation External validation (30% samples)	carnitine↑, creatine↑, TMAO↓	Diagnosis	HCC vs. LC Sensitivity 81% Specificity71%		Cutoff at 20 IU/mL HCC vs. LC Specificity 0%
Ladep/ 2014	Nigeria Gambia	Training set HCC, 63 LC, 32 Non-cirrhotic liver disease, 107 Healthy, 88 Validation set HCC, 141 LC, 56 Non-cirrhotic liver disease, 178 Healthy, 88	[1] H-NMR	PCA PLS-DA LR	Cross-validation External validation (independent cohort)	inosine↓, indole-3-acetate↑, NAA↑, galactose↑	Diagnosis	HCC vs. LC Training set AUROC 0.90 Sensitivity 86.9% Specificity90.3% Validation set AUROC 0.72 Sensitivity 77.1% Specificity 63.5%		HCC vs. LC Training set AUROC 0.68 Sensitivity 49.2% Specificity77.4% Validation set AUROC 0.58 Sensitivity 60% Specificity 66% BCLC-D vs. BCLC A-C not significant
							Prognosis (Clinical stages)	BCLC D vs. BCLC A-C (methionine, acetylcarnitine, indole-3-acetate, NAA, dimethylglycine, 1-methylnicotinamide, creatine) significant		
Shariff/ 2016	UK	HCC, 13 LC, 25	[1] H-NMR	PCA PLS-DA	Cross-validation	carnitine↑, formate↑, citrate doublet↓, hippurate↓, p-cresol sulfate↓, creatinine methyl↓, creatinine methylene↓	Diagnosis	HCC vs. LC Sensitivity 53.6% Specificity 96%		Cutoff at 20 IU/mL Sensitivity 45% Specificity 95%
Cox/ 2016	Bangladesh	HCC, 46 LC, 50 CH, 48 Healthy, 8	[1] H-NMR	PCA PLS-DA	Cross-validation	carnitine↑, creatine↑, TMAO↓, hippurate↓	Diagnosis	HCC vs. non-HCC carnitine↑, creatine↑, TMAO↓, hippurate↓ (P < 0.05)		HCC vs. non-HCC AFP↑ (P < 0.05)
Wang/ 2022	Animal (Rats)	HCC model, 18 Control, 18	[1] H-NMR	PCA		choline↑, taurine↑, creatinine↑, hippurate↓, PUT↑	Diagnosis	HCC vs. Control AUROC Hippurate: 0.812 (0.667–0.957) ^b^ creatinine: 0.701 (0.527–0.874) PUT: 0.738 (0.561–0.914) choline: 0.722 (0.547–0.897) taurine: 0.722 (0.551–0.894)		
Wu/ 2009	China	HCC, 20 Healthy, 20	GC-MS/MS	PCA	Cross-validation	octanedioic acid↑, glycine↑, L-tyrosine↑, L-threonine↑, butanedioic acid↑, other 13 metabolites↓	Diagnosis	HCC vs. Healthy PCA model of 18 metabolites AUROC 0.8825		HCC vs. Healthy AFP alone Cutoff at 20 ng/mL Sensitivity 75% [AFP + urinary metabolites] AUROC 0.9725
Li/ 2010	Animal (Rats)	HCC model, 5 HLM model, 5 Normal, 5	GC-TOF-MS/MS	PLS-DA		Serine↓, Glycine↓, 5-oxyproline↓, Malate↓, 2-methylsuccinic acid↑	Prognosis (Lung metastasis)	Completely separate HLM from HCC by PLS-DA	HLM vs. HCC Serum: serine, ornithine, phenylalanine, asparaginase, threitol, 5-hydroxyproline, 2,3,4-trihydroxybutyric acid↓; Lactic acid↑	
Chen/ 2011	China	Training set HCC, 55 BT, 16 Healthy, 47 Validation set HCC, 27 BT, 8 Healthy, 24	GC-TOF-MS/MS UPLC-QTOF-MS/MS	PCA PLS-DA	Cross-validation External validation	Not specified ^c^	Diagnosis	HCC vs. Healthy Accuracy 100% HCC (AFP < 20ng/mL) vs. Healthy Accuracy 100%		
Ye/ 2012	China	HCC, 19 Recurrent, 7 Non-recurrent, 11 Healthy, 20	LC-TOF-MS/MS	Binary LR		Ethanolamine↑, Lactic acid↑, Acotinic acid↑, Phenylalanine↑, Ribose↑	Prognosis (1-year recurrence)	Recurrent vs. non-recurrent accuracy 100%		
Osman/ 2017	Egypt	HCC, 55 LC, 40 Healthy, 45	GC-MS/MS	PCA		glycine↑, serine↑, threonine↑, proline↑, citric acid↑, urea↓, phosphate↓, pyrimidine↓, arabinose↓, xylitol↓, hippuric acid↓, xylonic acid↓, glycerol↓	Diagnosis	HCC vs. Healthy PCA model of 13 markers AUROC 1.00		
Shao/ 2015	China	Training set HCC, 33 LC, 27 Healthy, 26 Validation set HCC, 33 LC, 21	LC-QTRAP-MS/MS	PLS-DA Binary LR	External validation	carnitine C4:0↑, hydantoin-5-propionic acid↑	Diagnosis	Training set HCC vs. LC AUROC 0.786 Small HCC vs. LC AUROC 0.840 Validation set HCC vs. LC AUROC 0.773		Training set HCC vs. LC AUROC 0.778 Small HCC vs. LC AUROC 0.675 Validation set HCC vs. LC AUROC 0.528 Small HCC vs. LC Sensitivity 0%
Liang/ 2016	China	Training set HCC, 25 Healthy, 12 Validation set HCC, 15 Validation set, 10	LC-QTOF-MS/MS	PCA PLS-DA SAM	External validation	palmitic acid, alpha-N-Phenylacetyl-L-glutamine, phytosphingosine, indoleacetyl glutamine, and glycocholic acid ↓/↑ (not specified)	Diagnosis	HCC vs. Healthy AUROC 0.903 Sensitivity 96.5% Specificity 83.0%		
Dawuti/ 2022	China	HCC, 55 LC, 49 Healthy, 50	SERS	SVM	Cross-validation	adenine↓, guanine↓, deoxyribose↓, uric acid↓, uracil↓, proline, Urea, histidine, serine, tryptophan, alanine, creatinine: ↓/↑ (not specified)	Diagnosis	HCC vs. LC Sensitivity 79.6% Specificity 76.0% Accuracy 77.9% HCC or LC vs. Healthy Sensitivity 92.0% Specificity 77.8% Accuracy 87.0%		Serum AFP Sensitivity for HCC 34.5%

^a^ Only include the application of urinary biomarkers. ^b^ Ranges in parentheses represent the 95% confidence interval (95% CI). ^c^ No simplified diagnostic panel was provided. * Nations refer to the countries/regions of the tested patients, studies in animals are shown as the species of aexperimented animals

**Samples**: HCC, hepatocellular carcinoma. LC, liver cirrhosis. CH, chronic hepatitis. BT, benign tumors. **Platforms**: [1] H-NMR, proton nuclear magnetic resonance. GC-TOF-MS/MS, gas chromatography–time-of-flight mass spectrometry. UPLC-QTOF-MS/MS, ultra-performance liquid chromatography quadrupole time-of-flight mass spectrometry. SERS, surface-enhanced Raman spectroscopy. LC-QTRAP-MS/MS, liquid chromatography − hybrid triple quadrupole linear ion trap mass spectrometry. **Modeling and validation methods**: PCA, principal component analysis. PLS-DA, partial least squares discriminant analysis. LR, logistic regression. SAM, significance analysis for microarrays. SVM, support vector machine. **Biomarkers**: PUT, putrescine. TMAO, trimethylamine-N-oxide. NAA, N-acetylated amino acid. **Results**: AFP, alpha-fetoprotein. AUROC, area under the receiver operating characteristic

## Urinary proteins

### Transforming growth factor (TGF)

TGF-α is a single-chain polypeptide with three disulfide bonds and has a strong mitogenic activity on various cell types. In 1987, Yeh et al. determined the concentration of TGF-α in the urine of HCC patients via RIA and found it to be significantly elevated, with a sensitivity of 71.7%, superior to serum AFP. However, when combined with serum AFP, the sensitivity of urine TGF-α reached 93.5% [25]. On the other hand, TGF-β1 is a homodimeric polypeptide that stimulates cell growth and malignant transformation through autocrine mechanisms. TGF-β1 could be produced by HCC cells or tumor stroma and is mainly metabolized and cleared in the liver. Therefore, both the tumor size of HCC and the liver function could affect the level of TGF-β1 [26]. In 1997, Tsai et al. reported that an AUROC of 0.730 distinguishes HCC from liver cirrhosis, with a sensitivity of 53.1% and a specificity of 98.9%; when combined with serum AFP, the sensitivity increased to 84.0% and the specificity to 97.8% [17]. This study further proved that urinary TGF-β1 was an independent risk factor for HCC in a dose-dependent manner [odds ratio (OR) 1.08, 95% confidence interval (CI): 1.04–1.12] and was correlated with large tumor size (≥ 3 cm), diffuse growth pattern, and poor liver function. In addition, the levels of urinary TGF-β1 decreased significantly after transarterial chemoembolization (TACE), and hence were correlated with overall survival (OS) in HCC patients [27] (Fig. 5A). In summary, both urine protein markers are easy to test and have shown to be valuable for the diagnosis, treatment monitoring, and prognosis assessment of HCC. Nonetheless, no correlation has been established between the urinary TGF-α or TGF-β1 levels and serum AFP [17, 25]; both markers have a complementary diagnostic value in AFP-negative patients. However, follow-up studies for these markers are yet lacking.

### Urinary trypsin inhibitor (UTI)

UTI and its precursor, inter-α-trypsin inhibitor (IαI), are synthesized in the liver and excreted in the urine, exerting an anti-inflammatory role in inflammatory tissues [28]. In 2001, Noie et al. quantified urinary UTI levels in 61 patients who underwent partial liver resection (including 40 HCC patients). Urinary UTI increased early in the postoperative period and showed a correlation with serum concentrations of C reactive protein (CRP); the maximum increase (ΔuUTImax) was positively correlated with indocyanine green (ICG) clearance, indicating a liver function reserve and total operation time, while it was negatively correlated with resection rate [29]. These findings are consistent with the theory that urinary UTI is an acute-phase protein associated with residual hepatic functional reserve. In 2004, Lin et al. used ELISA and found that UTI levels in urine decreased with the aggravation of liver damage and were significantly lower in patients with hepatitis and liver cirrhosis than in normal controls but were slightly increased in HCC compared to liver cirrhosis [30]. The postoperative dynamics of urinary UTI are similar to those of serum CRP [29], suggesting that this molecule could be considered a monitoring parameter, albeit it lacks direct diagnostic and prognostic value in HCC.

### Neutrophil gelatinase-associated lipocalin (NGAL)

NGAL, also known as Lipocalin 2 (Lcn2), is a secreted glycoprotein that binds to a variety of hydrophobic molecules that endows it with critical transport functions, such as anti-infection immune response, the intra- and extracellular clearance functions [31], and the regulation of proliferation, invasion, and metastasis of cancer cells [32]. Zhang et al. revealed that the overexpression of NGAL and its cell surface receptor, NGALR, in HCC tissues is associated with poor pathological features and postoperative survival [33]. In the urine samples, Abdelsameea et al. quantified NGAL concentrations using ELISA and found that urinary NGAL levels increased with the progression of the disease from normal to chronic hepatitis to liver cirrhosis and HCC. Urinary NGAL could diagnose HCC from liver cirrhosis with an AUROC of 0.95, a sensitivity of 90%, and a specificity of 87.5%. When combined with serum AFP, the AUROC reached 99.7% [18]. Although urinary NGAL has shown diagnostic and prognostic value, it still lacks specificity for HCC [34]. Therefore, its potential in HCC screening is limited as current evidence only supports its complementary use for serum AFP.

### Matrix metalloproteinases (MMPs)

MMPs, especially MMP-2 and MMP-9, promote angiogenesis and tumor invasion by degrading the basement membranes composed primarily of type IV collagen [35]. Tissue- and serum-based studies have demonstrated the role of MMP-2 in promoting the progression of HCC [36, 37]. Suh et al. determined the levels of urinary MMP-2 and MMP-9 in HCC patients receiving radiotherapy and found that the levels of MMP-2 before radiotherapy were significantly correlated with recurrence and short progression-free survival (PFS) (Fig. 5B). The combination of urinary MMP-2 and serum vascular endothelial growth factor (VEGF)-to-platelet (PLT) ratio (VEGF/PLT) independently predicted poor prognosis (OR 2.12, 95% CI: 1.01–4.55) [38]. These results suggested that MMP-2 is a prognostic factor and a potential therapeutic target.

### Multiprotein models

Urine contains many proteins of various types, about 30% of which are derived from outside the urinary system [39]. With the development of testing platforms, urine-based proteomics has been widely used to screen for urological [40] and other malignancy markers [41, 42]. Several studies from various regions have explored urinary multiprotein models or proteomic markers for HCC [19, 43–46]. Abdalla et al. screened DJ-1, chromatin assembly factor-1 (CAF-1), and heat shock protein 60 (HSP60) as potential markers by LC-MS/MS in an Egyptian post-HCV HCC cohort. Quantitative reverse transcription PCR (RT-qPCR) confirmed the significant overexpression of the three corresponding genes. The overexpression of *CAF-1* and *HSP60* could diagnose HCC with a sensitivity of 61% and a specificity of 92% [19]. Huang et al. identified 83 upregulated proteins in HCC (mainly involved in signal transduction, inflammatory response, calcium ion binding, and other pathways) and 8 downregulated proteins (mainly tubulins). Further genomic, transcriptomic, and proteomic analysis of open datasets revealed the co-upregulation of S100A9 and GRN [47] (Fig. 5C-left and middle), the known promotors for HCC invasion and proliferation [48, 49]. Thus, diagnostic and prognostic panels for HCC were built with these markers [43] (Fig. 5C-right). Two studies from China proposed a random forest diagnostic model with 7 markers, and a quick-test qualitative diagnostic model with 2 markers, respectively. Both models showed excellent sensitivities and specificities of > 80% [44, 45]. In a cohort from UK, Bannaga et al. used capillary electrophoresis mass spectrometry (CE-MS/MS) to compare the protein profiles of HCC, liver cirrhosis, non-cirrhosis, and normal controls and identified 31 differential peptides. Then, a support vector machine (SVM) model, “HCC-31”, was established with an adequate diagnostic power for HCC in the validation set (AUROC 0.88, 95% CI: 0.81–0.93) [46] (Fig. 3A). In addition, *in silico* mapping deduced 5 upregulated proteases and 2 downregulated proteases, confirmed by immunohistochemistry (IHC) [46] (Fig. 3B). This might suggest future therapeutic targets against proteases that drive ECM remodeling, invasion, and spread of cancer cells [50]. Proteomic analysis has provided novel methods for HCC urinary marker screening. The present findings suggested several multiprotein models as diagnostic or prognostic tools, which have provided promising results validated through histological, genomic, and transcriptomic studies. Thus, it can be expected that many urinary protein markers for HCC would be identified in the future.

## Urinary nucleic acids

### DNA

As in other cancer types, DNA alterations are critical for initiating and progressing HCC. The DNA changes identified from the body fluids of HCC patients may provide novel biomarkers for the screening and early diagnosis of HCC [24]. Circulating free DNAs (cfDNAs) are DNA fragments of about 160 bp, mainly derived from cell phagocytosis and released into circulation [51]. As an essential component of liquid biopsies, cfDNAs reflect tumor genetic characteristics more comprehensively than traditional tissue biopsies [24]. Circulating tumor DNAs (ctDNAs) refer to the subset of cfDNAs that are directly derived from tumor cells. Urine is abundant with kidney-filtered low molecular weight DNAs (LMW DNAs) that can be used to identify DNA markers [51, 52]. Notably, the diagnostic sensitivity of DNA markers is expected to continue to increase due to significant advances in detection depth. However, considering the diverse etiologies, the signaling pathways involved in HCC, and the highly heterogeneous nature of cancer, combinations of several DNA markers from different pathways are preferred for diagnosis [24] (Fig. 4A).

To date, studies of urine DNA markers have mainly focused on several HCC-specific DNA mutations and methylations, including *TP53 249T*, *mRASSF1A*, *mGSTP1*, and *hTERT 124*. In 2011, Lin et al. first introduced the *TP53 249T* mutation, an HCC-specific mutation in the urine, detected in 9/17 patients but not in any of the controls [53]. Hann et al. demonstrated the potential of *mRASSF1A*, *mGSTP1*, and *TP53 249 T* for the early prediction of post-treatment recurrence during follow-up in 10 HCC cases. In 5 cases with tumor recurrence, all three DNA markers showed significant elevation prior to MRI confirmation. These markers could be positive for up to 9 months before MRI20 indicated recurrence. Wang et al. constructed a multifactor model by combining urinary *mRASSF1A*, *mGSTP1*, and *TP53 249T* and serum AFP that could distinguish HCC from hepatitis or cirrhosis with 87% sensitivity and 90% specificity, outperforming AFP alone. In addition, the present study compared different algorithms in the modeling process, including logistic regression (LR), classification and regression trees (CART), random forest (RF), and a two-step model combining LR with RF. RF and the two-step models proved to have the best AUROC and robustness [23]. Kim et al. established a diagnostic ctDNA panel in an international multicenter cohort based on the same three markers. The application of the ctDNA panel in AFP-negative patients significantly improved the diagnostic power of HCC to a sensitivity of 78.6% and a specificity of 90% (Fig. 4B). Specifically, this model increased the diagnostic sensitivity for early HCC from 40–77% [54]. Zhang et al. concluded that the positive rate of urinary *TP53 249T*, *CTNNB1 32–37*, *hTERT 124*, and *mRASSF1A* was significantly increased from hepatitis and cirrhosis to HCC [55]. *mRASSF1A* is the abnormal methylation of *RASSF1A*, a tumor suppressor gene from the *RAS*-associated domain family [56]. To further improve the specificity to HCC, Jain et al. compared the diagnostic power of methylation at different sites, revealing that P1 methylation had the best performance compared to E1 and P2 regions, and the sensitivity of P1 methylation of *RASSF1A* in AFP-negative HCC patients was up to 81.1% [56] (Fig. 4C).

### RNA

MicroRNAs (miRNAs) are non-coding RNAs with a length of about 22 nucleotides. Dysregulation of miRNAs has been linked to a variety of diseases, including cancers [57], thereby deeming them as appropriate tools for cancer management. The miRNAs may be actively released into circulation by microvesicle secretion or passively by apoptosis and necrosis [58] and filtered into the urine. The stability and resistance to endogenous RNase activity of miRNAs allow for the freezing and storage of samples, facilitating the development of urinary miRNA-based biomarkers [59]. Abdalla et al. screened for miRNA markers through the expression profiling of urine samples. Results showed that miR-625, miR-532, and miR-618 were upregulated, while miR-516-5p and miR-650 were downregulated in HCV-infected patients and post-HCV HCC patients. miR-618 and miR-650, the top two differentially expressed markers in RT-qPCR, together could diagnose HCC with an accuracy of 69% [60]. Similarly, Świtlik et al. identified miR-532-3p and miR-765 as a diagnostic panel that could stratify HCC patients into two prognostic groups with distinct histological classes, clinical stages, and metastatic status [61]. Zhou et al. identified miR-93-5p as a candidate biomarker by analyzing public sequencing datasets. The upregulation of miR-93-5p in tissues, plasma, and urine was confirmed in clinical samples (Fig. 6A–C). Urinary miR-93-5p could diagnose early HBV-related HCC with 87.5% sensitivity and 97.4% specificity, which was superior to serum AFP (Fig. 6D). One month after radical resection, urinary miR-93-5p decreased to normal levels (Fig. 6E). Moreover, the prognosis of patients with high urinary miR-93-5p levels was worse than in those with lower urinary miR-93-5p levels [16] (Fig. 6F).

## Urinary metabolites

### Polyamines

Polyamines are essential for the proliferation of normal and tumor cells. During the initiation and progression of HCC, the activity of guanylate decarboxylase is elevated, resulting in increased levels of polyamines [62]. In 1985, Kubota et al. reported that the urinary total polyamine levels were abnormally elevated in patients with various malignancies, including liver, gastrointestinal tract, and hematologic cancers, and decreased to the normal range after treatment [63]. In 1998, Antoniello et al. revealed a significant increase in urinary total, free, and acetylated polyamines in HCC patients using reversed-phase high-performance liquid chromatography (HPLC). Total putrescine (PUT), spermine (SPM), and spermidine (SPD) levels were significantly increased, among which PUT and SPD were mainly excreted in the acetylated form, while SPM was excreted in the free form [64]. Enjoji et al. reported that N [1], N [12]-diacetylspermine (DiAcSPM) could distinguish HCC from cirrhosis with a sensitivity of 65.5% and a specificity of 76.0%, but the efficacy in diagnosing early HCC was not significant. In addition, urinary DiAcSPM levels were significantly reduced after treatment [65]. Using ultra-HPLC-tandem mass spectrometry (UHPLC-MS/MS), Yu et al. quantified several polyamines and their metabolites in tissues, plasma, and urine in rat HCC models. Urinary N-acetylspermidine (NSPD), N-acetylspermine (NSPM), N [1], N [8]-diacetylspermidine (DiAcSPD), and DiAcSPM were significantly higher in the models than in the controls and decreased to the normal range after receiving anticancer drugs [66]. Studies by Enjoji et al. and Yu et al. suggested that polyamines might be useful as diagnostic and treatment monitoring markers in HCC. Yu et al. also compared the polyamines in tissue and body fluids and concluded that the synthesis of PUT and its metabolism to NSPD was enhanced in HCC. Moreover, the urine samples were sensitive for the detection of polyamine metabolites and potentially enriched with polar N-acetylated polyamines [66]. A similar conclusion was obtained by Liu et al. in an HCC patient cohort study, wherein NSPD, SPM, and SPD were significantly increased in the urine of hepatic cancer patients [67]. Nonetheless, whether polyamines are cancer-specific biomarkers is controversial. Hyltander et al. compared urinary polyamine levels in cancer patients and non-cancer patients undergoing major surgeries and minor surgeries, suggesting that the molecules are mainly associated with the metabolic stress of patients rather than cancers. Host factors, such as serum albumin concentrations, liver function, and liver metastases, might primarily determine altered excretion of polyamines in cancer patients [68].

### Nucleotides

The hypothesis that the balance between intracellular cAMP and cGMP may regulate cell growth, proliferation, and malignant transformation has been substantiated by in vivo and in vitro studies in various malignant tumors [68, 69]. Accumulating evidence suggests that increased cGMP or altered activity of guanylate cyclase are features of malignant tissues. In 1982, Dusheiko et al. observed that RIA-quantified urinary cGMP levels were significantly higher in HCC patients than in healthy controls, while cAMP levels were similar to those of controls. However, the findings were not specific to HCC, as dynamic changes in cGMP and cAMP were observed in other malignancies and patients with damaged liver functions [70]. The study by Turner et al. reached similar conclusions in cervical and breast cancer [71], suggesting that cyclic nucleotide metabolism is inclined to cGMP in malignant tumors [70, 71]. Urinary concentrations of nucleotides are determined by the balance of multiple processes, such as synthesis, degradation, and excretion, which might be affected by liver function status. These factors limit the clinical translational studies of urinary nucleotides as HCC biomarkers.

### L-Fucose

L-Fucose is located at the non-reducing end of the sugar chain of the conjugated sugar compounds. The diagnostic value of serum L-fucose in primary liver cancer has been reported as early as 1984 [72]. In 1990, Sakai et al. determined the concentration of L-fucose in the urine by biochemical methods and observed abnormally increased levels in patients with cirrhosis (19/21) and liver cancers (35/41), as well as in other diseases, such as gastric cancer, lung cancer, and gastric ulcer, suggesting a lack of specificity for HCC [73]. Currently, follow-up studies on urinary L-fucose are lacking.

### Volatile organic compounds (VOCs)

Dysfunctional cytochrome P450 may contribute to the progression of HCC [74]. The byproducts of cytochrome P450 include various VOCs. Based on this theory, Bannaga et al. identified seven VOCs between HCC and controls in the urine by GC-MS/MS, while the diagnostic model based on urinary VOCs distinguished between HCC and cirrhosis with an AUROC of 0.97 [75]. Bannaga et al. also established another multi-VOC diagnostic model by combining the solid-phase microextraction (SPME) technique with radial basis function networks (RBFN), which proved to be valuable for the diagnosis of a variety of cancers, including complementary diagnostic value for serum AFP in HCC [76]. However, the current research on VOCs is preliminary, and additional evidence is required to assess the clinical applications of such biomarkers.

### Multi-metabolite models

Tumor cells have specific metabolic characteristics, which could be represented by measuring the metabolites in body fluids [14]. The concept of “metabolomics” or “metabonomics” refers to the high-throughput analysis of metabolites in biological specimens. “metabolomics” focuses on the panoramic landscape of metabolites in samples, while “metabonomics” emphasizes the metabolic responses to pathological factors [77, 78]. Several recent studies have applied these methodologies to screen for urinary metabolite biomarkers. The main testing platforms include proton NMR ( [1] H-NMR) and MS [22, 79, 80] (Fig. 2A, B). Both methods have a complementary value to each other. MS has advantages in detection sensitivity, while [1] H-NMR has strengths in sample preparation and the reproducibility of the results [81].

Using [1] H-NMR, Cox et al. examined urine samples from hepatitis B, cirrhosis, and HCC patients and reported major differential metabolites, including upregulated carnitine and downregulated creatinine, hippurate, and trimethylamine-N-oxide (TMAO) in HCC [82]. Shariff et al. established a urinary multi-metabolite model for HCC via PCA and PLS-DA, and the sensitivity and specificity to distinguish HBV-related HCC from cirrhosis were 89.5% and 88.9%, respectively. The top contributing metabolites are upregulated creatine and carnitine and downregulated creatinine and acetone, which might be related to the changes in muscle mass, energy metabolism, and lipid metabolism [83]. A similar conclusion was derived in another cohort with HCV infection. Major differential metabolites include upregulated carnitine and creatine and downregulated TMAO, and the multi-metabolite model could distinguish between HCC and cirrhosis with a sensitivity of 81% and a specificity of 71% [84]. In a cohort with a heterogeneous etiological background, Shariff et al. identified increased carnitine and formate and decreased creatinine, hippurate, citrate, and p-cresol sulfate in the urine of HCC patients. Furthermore, the PLS-DA model showed a sensitivity of 53.6% and a specificity of 96% for diagnosing HCC, which is superior to serum AFP in the same cohort [85]. Ladep et al. revealed alterations in urinary metabolic profiles from non-cirrhosis liver disease to liver cirrhosis and HCC (Fig. 2C-left). The diagnostic panel composed of inosine, indole-3-acetate, N-acetylated amino acid (NAA), and galactose can distinguish HCC from cirrhosis with an efficacy better than serum AFP. In addition, the urinary metabolic markers were significantly associated with clinical stages [86] (Fig. 2C-right). Similarly, Wang et al. observed the separation of metabolic profiles between HCC rat models and controls, which was parallel to the progression of HCC. Pathway enrichment analysis indicated that taurine and hypotaurine metabolism was involved in HCC [80].

Using GC-MS/MS, Li et al. detected urinary metabolic alterations from controls to HCC and HCC with lung metastasis (HLM) in rat models. Downregulated serine, glycine, 5-oxyproline, and malate and upregulated 2-methylsuccinic acid levels were observed in HLM samples compared to HCC samples. The multi-metabolite model can accurately distinguish between HCC and HLM models [87]. In clinical cohorts, the PLS-DA and PCA multi-metabolite models established by Chen et al. and Osman et al. distinguished between HCC and healthy controls with excellent accuracy superior to serum AFP [8, 88]. Wu et al. established a PCA diagnostic model combining serum AFP to 18 urinary metabolites; the AUROC for diagnosing HCC reached 0.9725 [79]. These studies suggested the potential of urinary metabolites in the screening and surveillance of HCC as supplementation to serum AFP. Regarding the prediction of postoperative recurrence, Ye et al. determined a prognostic model including ethanolamine, lactic acid, acotinic acid, phenylalanine, and ribose, which could distinguish between HCC patients with and without recurrence with an accuracy of 100% [89]. Notably, this result needs external validation in large cohorts. Based on LC-MS/MS, Liang et al. screened 15 differential metabolites between HCC patients and healthy controls (Fig. 2D-left). The significantly altered pathways included bile acid biosynthesis, the citric acid cycle, tryptophan metabolism, and the urea cycle (Fig. 2D-right). A model involving 5 metabolites was selected via significance analysis for microarrays (SAM), which showed an AUROC of 0.903, a sensitivity of 96.5%, and a specificity of 83% in diagnosing HCC [90]. Shao et al. developed a pseudo-targeted detection method based on liquid chromatography-hybrid triple quadrupole linear ion trap mass spectrometry (LC-QTRAP-MS/MS), which combines good signal quality and detection sensitivity [91]. Carnitine C4:0 and hydantoin-5-propionic acid were selected to build a diagnostic panel that detects early HCC with an AUROC of 0.773 in external validation [92].

Other techniques, such as surface-enhanced Raman spectroscopy (SERS), have also been utilized to analyze the biochemical fingerprints in body fluids [93]. Dawuti et al. identified several dysregulated metabolites of nucleotides and amino acids using SERS. The SVM model along these metabolites could distinguish HCC from cirrhosis with a sensitivity of 79.6%, while the sensitivity of serum AFP was only 34.5% in the same cohort [94].

Metabolomic or metabonomic studies have recently become a hot research topic. The multi-metabolite models derived from the current studies seem promising for managing HCC, especially for the discrimination between HCC and liver cirrhosis [83, 84, 92, 94]. In addition, the consistency of the results from distinct testing platforms, regions, and etiological backgrounds further supports the interpretability, universality, and applicational value of these biomarkers. Interestingly, some studies have shown an overlap between HCC cirrhosis metabolic difference and cirrhosis healthy metabolic difference [82–84, 92, 94] (Fig. 2C-left). Additionally, animal experiments have shown a progressive shift of metabolic profiles parallel to the development of HCC [80, 82], while other studies can significantly distinguish between HCC and healthy people but not between HCC and cirrhosis [88]. Taken together, these findings suggested that the metabolic biomarkers may reflect the biological behavior of malignant tissues and are influenced by the background hepatic lesions. These conclusions were in line with the biological and clinical features of HCC. On the other hand, these findings suggested that the specificity of the metabolic markers in diagnosing HCC from cirrhosis patients should be under intensive focus.

## Other urinary biomarkers

The markers mentioned above were primarily developed in HCC cohorts or HCC animal models to provide a direct value for the clinical management of HCC. Some studies focused on urinary markers associated with exposure to aflatoxin and inflammatory oxidative stress; both are essential factors in the pathogenesis of HCC. These markers may contribute to prevention, screening, and surveillance of HCC in high-risk populations.

### Urinary aflatoxin and its metabolites

Aflatoxin exposure is a widely acknowledged risk factor of HCC [1, 2]. Significant concentrations of several aflatoxin derivatives in serum and urine, such as aflatoxin-albumin adducts and aflatoxin-N7-guanine adducts, have been associated with aflatoxin-DNA adducts in liver tissues, suggesting a potential value for the assessment of aflatoxin exposure [95]. In a prospective cohort, Ross et al. quantified urinary aflatoxin B1 (AFB_1_) and aflatoxin metabolites, including AFP_1_, AFM_1_, and aflatoxin DNA adducts AFB_1_-N [7]-Gua, which were markedly elevated in HCC patients. The presence of any of these compounds was an independent risk factor for HCC [relative risk (RR) 3.8, 95% CI: 1.2–12.2], and AFP_1_ showed the highest RR of 6.2 (95% CI: 1.8–21.5). In addition, a significant risk was associated with urinary aflatoxin products in the HBsAg-positive group [21]. Also, the synergistic risk effect of serum HBsAg and urinary aflatoxin metabolites was observed by Wang et al. [96]. A cross-sectional study revealed that the average levels of urinary aflatoxin metabolites in random volunteers were positively associated with average HCC mortalities in the same county [97]. Although the contribution of these studies to clinical precision medicine is not direct, aflatoxin-related biomarkers may play roles in the prevention, screening, and surveillance of HCC.

### Urinary biomarkers associated with oxidative stress

Chronic inflammation, continuous damage, and regeneration of liver tissues are the common pathological processes in HCC with different etiological backgrounds [98]. Oxidative stress is the imbalance between exogenous and endogenous reactive oxygen species (ROS) and the anti-oxidant function. Excessive ROS can directly mediate lipid peroxidation and DNA damage and promote the progression of liver disease and liver cancers [99]. Therefore, oxidative stress-related metabolites have the potential to serve as the markers of cancer risk, especially in populations with a known background of chronic inflammatory liver diseases, such as chronic hepatitis and liver cirrhosis. Nair et al. quantified urinary etheno-deoxyadenosine (ε-dA), a DNA-reactive aldehyde produced by the reaction of DNA with lipid peroxidation products and found that ε-dA levels were 20–90 times higher in patients with HCC, cirrhosis, or chronic hepatitis compared to asymptomatic HBV carriers [100]. In a large follow-up cohort, Yuan et al. revealed significantly elevated urinary 8-epi-Prostaglandin F2α (8-epi-PGF2α), a product of lipid peroxidation, in patients who developed HCC compared to the controls. The group of patients with the highest quartile of 8-epi-PGF2α levels had a RR of 2.55 (95% CI: 1.62–4.01). In addition, a significant increase could be detected as early as 10 years before the diagnosis of HCC [101]. Ma et al. identified urinary 15-F_2t_-isoprostane (15-F_2t_-IsoP) as a risk factor for HCC. The group with the highest quartile of 15-F_2t_-IsoP levels had an OR of 1.75 (95% CI: 0.70–4.42) in females and an OR of 8.84 (95% CI: 2.74–28.60) in males [102]. In addition, Wu et al. reported a synergistic risk effect of urinary AFB_1_, 15-F_2t_-IsoP, and 8-oxo-7,8-dihydro-2’-deoxyguanosine (8-oxodG), markers of oxidative stress, suggesting that 15-F_2t_-IsoP may also serve as a marker for aflatoxin exposure [103].

## Conclusions and outlook

This review summarized a series of urinary biomarkers of different molecular types and their application in the screening and surveillance, diagnosis, treatment, monitoring, and prognosis of HCC. Next, we compared each marker from the “starting point”, i.e., its cohort information, detection platform, and modeling method, to the “endpoint”, namely its direction of dysregulation, diagnostic power, and prognostic power. The included studies are mainly from East Asia and Africa, which is in line with the significant disease burden of HCC that needs to be addressed in these regions. The subjects of these studies included HCC patients with diverse backgrounds, including HBV and HCV infection, aflatoxin exposure, and NAFLD, which was conducive to generalizing the current conclusions. Most of the reviewed studies have set control groups comprising patients with chronic hepatitis and liver cirrhosis for HCC screening; early HCC in patients with cirrhosis is a prominent challenge in managing HCC.

The analysis of urinary biomarkers for HCC has shown an increasing trend in recent years. The significant advances in detection methods and analytical algorithms would facilitate the future detection of many molecular markers. Notably, many of these are early-stage studies, including animal experiments and preliminary analysis of raw data, and are still far from clinical application, which requires simplified diagnostic models and easy detection techniques. Nevertheless, many researchers have improved the reliability of their conclusions by validation in independent cohorts using serum and tissue samples and analysis compared to or in combination with serum AFP.

Although urinary biomarkers provide promising tools for solving the bottleneck problems in managing HCC, future research and clinical translation must overcome several challenges. First, a simple method should be established to determine the biomarkers in urine quantitatively. Second, the sensitivity and specificity of the biomarkers should be validated in large, independent, and prospective cohorts. In addition, the specificity toward HCC should be further demonstrated, especially after adjusting the influence of liver dysfunction, background liver lesions, and secondary homeostasis disorders. Therefore, additional studies are needed to investigate the biological functions and molecular interactions of these biomarkers.

## Data Availability

Data availability does not apply to this article as no new data were generated or analyzed in this study.
